# Invited reply: Comment on ‘Left–right asymmetry of the Maxwell spot centroids in adults without and with dyslexia’

**DOI:** 10.1098/rspb.2024.3036

**Published:** 2025-02-12

**Authors:** Albert Le Floch, Guy Ropars

**Affiliations:** ^1^ Laboratoire de Physique des Lasers, UFR SPM, Université de Rennes, Rennes 35042, France; ^2^ Laboratoire d’Electronique Quantique et Chiralités, 20 Square Marcel Bouget, Rennes 35700, France

**Keywords:** human vision, asymmetries, noise effects, neurological disorders

The aim of our 2017 paper [1] was to try to identify the ‘symmetry breaking’ that occurs somewhere in the visual system of normal readers leading to their ocular dominance. Such an asymmetry was suspected by Ernst Mach himself around 1900 [2]. We thought we had identified the necessary asymmetry by comparing the profiles of the two Maxwell centroids at the centre of the foveas of normal readers. Conversely, we observed a lack of asymmetry in people with dyslexia. Recently, a comment by Naudet, Seidenberg and Bishop [3] raised some questions about our 2017 paper. These questions give us the opportunity to clarify some features and highlight some points that have escaped their attention.

In their summary (page 1) of our work, Naudet *et al*. reduced our 2017 proposal to ‘a novel version of a visual theory of dyslexia’ within the framework of theories proposing a ‘purely visual basis for dyslexia’. Of course, vision is essential for reading, but the lack of asymmetry observed in our model for 90% of the dyslexics (27/30 as Naudet *et al*. write in table 1 [3]) essentially induces perturbations in the connections of different regions in the brain, such as mirror images through the corpus callosum (fig. 5 in the 2017 paper), including the multimodal areas. Indeed, as shown by many groups over the last few decades, the integration of converging inputs from many senses is indeed well established from birth in cross-modal areas [4–9]. The symmetric and non-symmetric interhemispheric projections through the corpus callosum are crucial for the justification of the mirror images and the duplicated images respectively [10]. Furthermore, the retina is a part of the central nervous system [11,12].

Concerning the two experimental methods introduced in the 2017 paper, we agree that the foveascope is a new device of our own conception. It is designed to permit the very small outlines (100–150 μm diameter) of the entoptic Maxwell centroids to be enlarged and recorded. This small area, at the centre of the foveas, with no blue cones, is important because it gives the human eye its greatest acuity, as it is the only area without the large chromatic dispersion in the blue introduced by Newton. The foveascope allows the outlines of the Maxwell centroids to be accurately recorded and facilitates the measurement of their relative asymmetry.

Regarding the second method concerning the observation of the noise-activated negative afterimages, Naudet *et al*. write that ‘the dominant eye is defined as the one where the afterimage takes longer to fade’. They may have been confused about the technique we used in the noise-activated afterimage dominant test. In fact, we did not use the fading time but the relative luminance and quality of the two negative afterimages, as shown in figs 2 and 4 of the 2017 paper.

Regarding the expressions ‘first cohort’ and ‘second cohort’ in our original 2017 paper, we agree that these two expressions were ambiguous. The two cohorts were not tested sequentially. We did not test the 30 controls first and then the 30 dyslexics. The 60 students from our university were tested over 2 years, according to their availabilities. Concerning the modulation frequency between 0.1 Hz and 1 Hz for the exchange blue-green filter, the comments by Naudet *et al*. give us the opportunity to clarify that each observer optimizes the frequency to improve the contrast to his or her best convenience. The chosen frequency has no effect on the geometry of the outline itself which remains the same over several observations.

Mirror reading errors associated with the left–right confusion do not seem to be a unique and rare case. For example, in his 2018 paper, Corballis [10] cites references to observers with dyslexia, and Simpson [13] reports the common ‘was-saw’ confusion in dyslexia. Based on the cohort of 30 dyslexic students, Naudet *et al*. consider that our conclusion overinterprets the findings of the study. However, these findings were supported by a second cohort of 160 children with dyslexia in which 60% of children perceived mirror images and 35% perceived duplicated images [14], inducing an internal visual crowding. Furthermore, the recent eye tracker experiment during silent reading [15] demonstrates the erasing of mirror images similar to that shown in fig. 5 in our 2017 paper. In fact, the excess of eye fixations and longer reading times induced by the *internal* visual crowding have also been observed in many laboratories during reading tests with dyslexic readers [16–18] and can be cancelled by simply switching to the pulsed light mode [15]. Moreover, an *external* crowding has previously been observed especially in children with dyslexia, which can be reduced using larger letter spacing [19–21].

In conclusion, the results of the six figures of our 2017 paper and of the three figures of the electronic supplementary material have not been questioned by Naudet *et al*. nor discredited to the best of our knowledge. In our 2017 paper, we have shown the existence of the Maxwell centroids and that they can be recorded for any observer. For a typical reader, an asymmetry between the outlines of the Maxwell centroids is seen, which determines the ocular dominance, while, for 90% of dyslexics, a lack of asymmetry has been shown to induce perceived mirror or duplicated images, i.e. a noisy *internal* visual crowding which, however, remains controllable [15].

We hope we have cleared up any inaccuracies and misunderstandings so that our results can be used with confidence as a basis for future work.

## Data Availability

This article has no additional data.
